# Surgery

**Published:** 1896-11

**Authors:** John Parmenter

**Affiliations:** Professor of anatomy and adjunct professor of surgery, Buffalo University Medical College


### Progress in Medical Science.

### SURGERY.

By JOHN PARMENTER, M. D.,

Professor of anatomy and adjunct professor of surgery, Buffalo University Medical College.

KELLY (*New York Polyclinic*, August 15, 1896,) describes his method of treating burns. He regards the inefficient results obtained so often as due to the nonrecognition of asepsis in the management of this class of cases. Indeed, he would make asepsis the fundamental principle in the therapy of burns. He mentions the dry dressing to condemn it as impracticable, for the reason that asepsis cannot be maintained and that toxic effects are prone to follow. The author's method (for which he claims no originality) is simply a moist dressing, for which he claims the following advantages—namely, that "the materials are all easy of procurement, easily sterilised or impregnated with antiseptics, if required, and are easy of application." Furthermore, the dressing is most efficient in controlling or at least mitigating sepsis, is comfortable to the patient when applied, and its removal is absolutely painless. Asepsis is preferable to antisepsis. Shock is met by using morphia hypodermatically. The wound is first irrigated with sterile water (110°) or Thiersch's solution if there be no sepsis present; with bichloride solution (1-1000) where infection has already occurred, to be followed with sterile water when the surface irrigated is large. Unbroken blisters are never ruptured, as the epidermal covering protects the surface beneath from infection. When tense and threatening rupture they are aspirated subcutaneously with a sterile needle. The dressing proper is adjusted to the different degrees of severity; portions simply hyperemic are anointed with a 5 per cent. solution of phenol in sterile olive oil, which reduces heat and itching when present; strips of gutta-percha tissue previously kept in a 2 per cent. carbolic acid solution. This same material may be applied over unbroken blisters to protect them from rupture. Over areas where slough is impending a moist gauze compress is applied, covered with rubber tissue to prevent evaporation. Over all a large thick layer of sterile cotton is laid and held in place with a well-fitting bandage.

The author at this juncture emphasises the necessity for the same scrupulous cleanliness of hands and instruments as in any aseptic operation, and the maintaining of these precautions throughout the conduct of the case. He advises changing the dressings daily during the first week, after which time every second or third day will suffice. As to iodoform, he regards it as useful but dangerous when applied over extensive surfaces. Grafting may be done after the separation of the sloughs. Where deep losses of substance have occurred, plastic operation by flap with pedicle may be best employed. Few cases require much more than a month for their satisfactory completion.

---

NANCREDE (*Annals of Surgery*, August, 1896,) contributes an article upon the operative treatment of Jacksonian and focal epilepsy, in which he relates the histories of four cases, in three of which a traumatism and in a fourth a probable apoplectic effusion were the causes producing the epileptic attacks. The final results in these cases, as well as in others, similarly caused and operated upon, leads the author to make the following conclusions :

(1) Removal of the discharging lesion in cortical and Jacksonian epilepsy can only be regarded as palliative, the operative scar in all instances thus far accessible to me, in time, becoming a new source of irritation. (2) The earlier the operation is done after the disease becomes fully established, the longer will the immunity last, and it is possible that if trephining is done very early, the operation may in a few instances prove curative, especially if any reliable method can be devised to lessen the extent of the inevitable scar and adhesions between the brain and the membranes. (3) That operation is not so dangerous in competent hands as to forbid our urging trephining in this class of epilepsies, especially when done early, because the chance of prolonged immunity is great, and the fits are apt to be lighter and to recur at greater intervals after relapse than before trephining. (4) Removal of the discharging lesion is imperatively demanded as a life-saving measure in those rare cases where the intervals between the fits are so short that the paroxysms are practically continuous. (5) In all cases, but especially those characterised by frequent paroxysms, it is an error in practice to permit the early resumption of work, particularly manual labor. Thus, in addition to the last case cited, I would call attention to another where I trephined for ordinary traumatic epilepsy, which remained perfectly well for nearly two years, until attempting to lift a heavy weight, the encephalon becoming suddenly congested, the patient at once had a fit, since when the convulsions have been nearly as frequent as they were before operation. (6) Operation removes only one of the factors productive of epilepsy, but the early response to inadequate stimulation remains, and can only disappear, if ever, after a prolonged period ; therefore, careful avoidance of everything which can, either through the mind or body, excite sudden and severe acute cerebral congestion or undue, prolonged, mental strain, constant congestion of the nervous centers, must be avoided for the longest practicable period—for the remainder of life, if possible.

---

Wirt (*International Journal of Surgery*, September, 1896,) details his treatment of chronic joint affections with dry heat at high temperature. This means the prolonged use of a temperature of 250° to 300° F. for a period of one-half to one and one-half hours. For this purpose an apparatus was constructed, which consists of a copper cylinder twelve and one-half inches long and ten inches in diameter. Fitting into each end of the cylinder is a wooden ring or disc, one inch wide by one inch thick. The wooden rings are secured in the ends of the cylinder by eight short screws passing from the outside through the cylinder into the rings. At each end of the cylinder is a hood, which encircles the limb and is drawn tight by means of puckering strings. The hood is made by double-coated rubber cloth, and is attached to the cylinder by being nailed to the wooden rings, the extreme end of the hood being held in place by a wire band. On each side of the cylinder, diametrically opposed, are two or three holes one-half inch in diameter. The purpose of these holes is to allow a rapid change of air in the cylinder. The cylinder is supported horizontally in a wooden frame, so that the lower surface of the cylinder is eighteen inches from the floor, or about the height of an average chair. Heat is applied by means of oil, gasoline, or the gas jet and the mixed gas and air jet, which is the Bunsen burner; the latter is probably the most convenient. The heat should not be turned on too rapidly at first, as the patient's endurance is greater if the heat is applied gradually.

The author describes his method for treating the knee as follows:

I apply a layer of cotton to the back of the limb in the popliteal region, securing it in place by loosely tied tapes. This is done for two reasons: in the first place, it will equalise the temperature, as undoubtedly the lower part of the cylinder is much hotter than the upper, and secondly, I found that the profuse perspiration resulting from the high temperature caused a dropping of fluid to the bottom of the cylinder, where it was instantly turned into steam, and this steam would immediately scald the limb. The limb being thus protected by the cotton, is inserted into the cylinder until the knee is in the center, the heel placed on a chair high enough to keep the calf from touching, if possible, even the wooden rings, which, though they do not ordinarily burn, yet may get uncomfortably warm. The hoods are drawn tightly around the limb, and at the start rubber corks are inserted in the holes in the cylinder, but are withdrawn when the cylinder is thoroughly heated. The heat is then applied to the cylinder and is carried up to the limit of endurance of the patient, which varies from 250° to 300°, and is kept at this point for an hour or more. One patient of mine could stand a temperature of 290° for over an hour. In a case I recently treated, I carried the temperature to the extreme point of 360° F., for a period of nearly five minutes.

The thermometer I use is a laboratory thermometer with a scale that will register up to 450° F. I have found it most satisfactory to allow the patient to hold one end of the thermometer while the bulb end is held within the cylinder alongside the knee against the cotton. Care should be taken not to touch the limb with the thermometer, for if this happens the limb will be blistered; this can easily be prevented by the proper use of the cotton.

The effect of such a high temperature is to enormously increase the perspiration from the local part, to increase perspiration over the whole body, to increase the amount of blood to the part, or at least the surface circulation, to increase the lymphatic circulation, to relieve pain and to increase mobility in the joint. The relief of pain and the increased mobility last for some hours after the application of the heat.

---

HEART-STRAIN—Pawinski regards caffeine as of especial use in functional and degenerative disease of the heart-muscle, and especially in the early stages. Sudden heart-strain from emotion or during fevers is particularly benefited by caffeine.—*Medical Times and Hospital Gazette.*

